# Maize Silage Kernel Fragment Estimation Using Deep Learning-Based Object Recognition in Non-Separated Kernel/Stover RGB Images

**DOI:** 10.3390/s19163506

**Published:** 2019-08-10

**Authors:** Christoffer Bøgelund Rasmussen, Thomas B. Moeslund

**Affiliations:** Department of Architecture, Design & Media Technology, Aalborg University, Rendsburggade 14, 9000 Aalborg, Denmark

**Keywords:** deep learning, object recognition, precision agriculture, silage, kernel processing, forage

## Abstract

Efficient and robust evaluation of kernel processing from corn silage is an important indicator to a farmer to determine the quality of their harvested crop. Current methods are cumbersome to conduct and take between hours to days. We present the adoption of two deep learning-based methods for kernel processing prediction without the cumbersome step of separating kernels and stover before capturing images. The methods show that kernels can be detected both with bounding boxes and at pixel-level instance segmentation. Networks were trained on up to 1393 images containing just over 6907 manually annotated kernel instances. Both methods showed promising results despite the challenging setting, with an average precision at an intersection-over-union of 0.5 of 34.0% and 36.1% on the test set consisting of images from three different harvest seasons for the bounding-box and instance segmentation networks respectively. Additionally, analysis of the correlation between the Kernel Processing Score (KPS) of annotations against the KPS of model predictions showed a strong correlation, with the best performing at r(15) = 0.88, p = 0.00003. The adoption of deep learning-based object recognition approaches for kernel processing measurement has the potential to lower the quality assessment process to minutes, greatly aiding a farmer in the strenuous harvesting season.

## 1. Introduction

Maize kernel processing evaluation is an important step in determining the quality of silage harvested from a forage harvester. Maize silage is used as fodder for cattle in dairy production and high quality silage though correct processing has an effect on milk yield [1] and suboptimal setting of the machinery can also lead to the quality being affected by up to 25% [2]. Kernels must be sufficiently cracked for efficient starch intake by lowering the requirement for chewing during eating and ruminating [3]. Kernels are processing by two mill rolls which compress and shear the plant. The gap known as the Processor Gap (PG) is often between 1 and 4 mm with 0.1 mm increments. This work focuses on the evaluation of kernel processing for silage quality efficiently through deep learning computer vision based methods via Convolutional Neural Networks (CNNs). Currently, the particle size distribution of kernel processing is evaluated through means which can be time consuming, cumbersome to conduct, and prone to error. An example of this is the Corn Silage Processing Score (CSPS) [3] and is one of the major standards in kernel processing evaluation. CSPS gives an analytical measurement of the kernel processing though laboratory equipment situated offsite typically returning a measurement after a number of days. In CSPS the user places a 160 g dried sample of harvested silage on a Ro-Tap sieving system which oscillates to allow processed kernels to pass through a number of differently sized sieve screens. The materials that pass through a 4.75 mm sieve can be measured for starch content and the percentage of this that passes is the CSPS. Particles larger than this size may result in a slow starch digestion in cattle and increase chewing requirement. The CSPS can be interpreted according to [3] as greater than 70% is optimal processing, between 50% and 70% is adequate processing and less than 50% is considered inadequate processing. An additional finer sieve screen of 1.18 mm can be used to determine the number of over-processed kernels. The starch content in such fragments can simply pass through the cow’s rumen, leading to wasted plant.

Another commonly used method for assessing kernel processing is the Penn State Particle Separator (PSPS) [4]. PSPS is similar to CSPS, however, does not require off-site laboratory equipment such as the Ro-Tap system or drying of the silage before starting the measurement process. Therefore, PSPS is able to give a farmer a much quicker indication of the kernel processing from the forage harvester. In PSPS three or four stacked trays with varying gaps are used to separate the kernel particles. The sample is placed in the top tray and the stack is shook a total of 40 times at a rate of one shake per second. After this, the weight of each tray is measured and is used to determine the distribution of kernel processing in the sample. Despite PSPS being more flexible than CSPS, the method is sensitive to the rate of shaking and moisture content, potentially giving a less accurate measurement.

The water separation method [5] can also be an effective method for a farmer to conduct a quick assessment of the kernel processing. Here, the total number of whole kernels in a 1-quart (946 mL) sample is evaluated. If more than one whole kernel per quart is found, the kernel processing is deemed not optimal. The method begins by placing the sample in a container filled with water. Then the sample is stirred gently until the stover, such as leaves and stalks, float and the kernels sink. Afterwards the stover and water is removed from which the number of whole kernels can be counted.

As mentioned, the aforementioned current kernel processing assessment methods are relatively time-consuming and can require potentially error-some manual steps. There has been minimal work done in automating this process and to our knowledge only one such exists. In this work computer vision is used to calculate the kernel particle size distribution [6]. In the method, first kernels must be separated from the stover using a method such as water separation. After this, the kernels are placed without touching any other samples on a dark background together with a common coin whose size is known, such as a penny. The coin can then be used as a reference later on in the system to calculate the kernel sizes. An image is captured and the contours of the kernel particles are found via image processing. Then the maximum inscribed circle is found for each particle in pixels which is converted to a kernel particle size distribution in millimetres. Metrics such as the percentage of particles smaller than 4.75 mm or average area give an indication to the user of kernel processing quality.

Looking into the broader domain, there is a large amount of research into measuring the quality of other crops. Firstly, the grades of product are determined by calculating rice kernel shape and size features and training a support vector machine [7,8]. Additionally, in [9] rice colour features and Fourier descriptors for shape and size are extracted from which the quality grade is determined through multivariate statistical analysis. A number of methods identify whole or broken fragments in grains. In [10], the size, color and brightness values are used in combination with a flatbed scanning device. In [11], rice is segmented based on color, and shape features indicate the grade of the crop. Classification of the grains in the image can be necessary when different grain types are mixed. Artificial neural networks have been used to classify types based upon extracted handcrafted features. In [12] color and texture features, in [13] size, color, and shape features, and in [14] color and morphological features were used to train networks respectively. K-Nearest Neighbor classifiers were trained on size and texture features in [15,16], with a number of color models being used in the latter. The quality of maize seeds was evaluated in [17] using hyperspectral imaging where data was reduced through t-distributed stochastic neighbourhood embedding and Fischer’s discriminant analysis for quality classification.

The works mentioned so far all follow that traditional computer vision approach of extracting hand-crafted features followed by using a classifier to make a decision on the task at hand. However, since 2012 when AlexNet [18] won the ImageNet classification challenge by a significant margin, deep learning with CNNs has dominated the field. Object recognition in images is a challenging task due to potential variations in objects, such as the colour, texture, shape, and size, and variations in images, such as the lighting, viewpoint, and occlusion. CNNs have been shown to learn complex patterns in data through a hierarchy of layers. Typically earlier CNN layers capture simple patterns such as the edges, while later layers learn more complex representations such as the shape of specific objects. This hierarchy has the potential to learn a powerful model given high quality data. There are numerous examples of machine vision with deep learning in agriculture that show good results and in many cases a significant improvement over using hand-crafted features. Examples include [19], where fully convolutional neural networks were trained to predict a semantic segmentation map of clover, grass, and weeds in RGB images containing clover-grass mixtures to estimate the distribution of the classes in the field. Here, they account for the potentially large amount of training data required for CNNs, as it was observed the annotation could take up to 3.5 h for 10 images. New images were simulated by combining augmented objects from those already annotated on top of captured background images. A deep learning approach to detect tomato plant diseases and pests was done in [20], where a number of popular models was evaluated for the task. In [21] a CNN and random forest classifier was trained to classify 32 different species of leaves. Plant disease detection of 14 different crop species including 26 diseases was done in [22] using CNNs and a number of different feature extractors such as AlexNet [18]. Crop and weed detection using CNNs was done in [23] on a combination of RGB and near-infrared data.

The aim of this work is to create a system to localise kernels fragments in RGB images for kernel processing assessment without the requirement separation of stover and kernels such as in [3,4,6]. Such a system will allow the farmer to gain an insight into the quality of the kernel processing without the need to perform a time-consuming and cumbersome process. We propose to train CNNs in both a bounding-box detector and instance segmentation form to automatically detect and localise kernel fragments in the challenging images. Examples of the images used in this work are shown in the following section in Figure 3. The methodology in training the aforementioned networks will be covered in Section 2 and the achieved results in Section 3.

**Figure 1:**
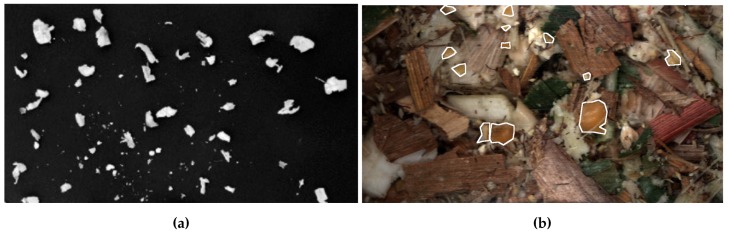
Example of the difference in images between separated and non-separated corn silage. (**a**) Reprinted from [6], with permission from Elsevier; (**b**) Example image from this work.

An example of the difference between separated kernel/stover images such as those typically used in [6] and non-separated used in this work can be seen in Figure 1. Additional white outlines in Figure 1b represent the outline of kernel fragments.

## 2. Materials and Methods

This section details the materials and methods used in the work. This includes images, subsequent kernel annotation and overview of the CNN models and training parameters. In order to train the respective recognition algorithms, a dataset of harvested silage is required. Both the basis for the images of the silage and annotation with resulting datasets is covered.

### 2.1. Images

**Figure 2:**
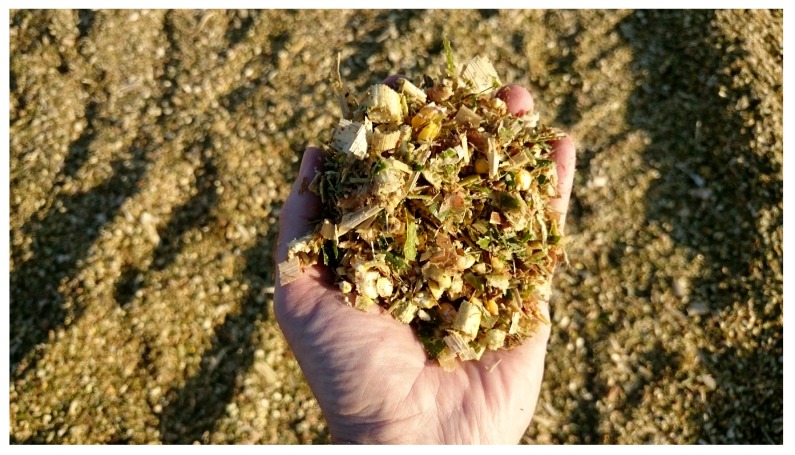
Example of harvested silage.

**Figure 3:**
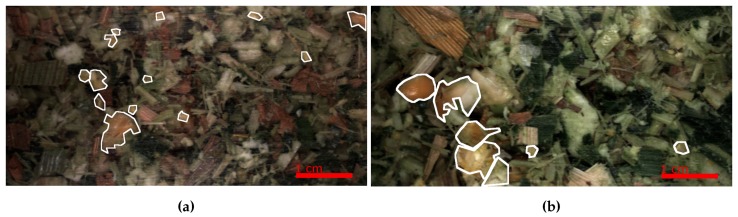
Example images of the differences in silage harvested with varying fragmentation. The white outline shows kernel fragment annotation outlines. (**a**) Smaller Processor Gap (PG) resulting in smaller kernel fragments; (**b**) Larger PG resulting in larger kernel fragments. A scale in the bottom right of the images shows the size of the images where 200 pixels is equal to 1 cm.

RGB colour images were taken of harvested silage over three years. The silage was produced from a variety of fields and crop conditions, and harvested with different machine settings. For example, the PG primarily accounts for the differences in the level of kernel fragmentation by altering the distance between two rollers mills in which the corn plant passes through. Secondly, the cutting length (CL) affects how fine the corn plant is chopped before passing through the rollers. Figure 2 shows an example of harvested silage, while the two images in Figure 3 show the differences in the harvested silage and a small PG (a) and a larger PG (b), resulting in a higher proportion in smaller and larger kernel fragments respectively. The silage in both (a) and (b) were harvested with same CL. Additionally, in the images a scale is shown in the bottom right indicating 1 cm, which equates to a resolution of 0.05 mm per pixel.

### 2.2. Datasets

The images were annotated using a tool with user defining vertices outlining the kernel’s fragments creating a polygon for each instance in a given image. These vertex-based annotations can be used to train both the instance segmentation models or they can be converted to bounding-boxes by taking the outer extremas of the annotated vertices for detection models. Just under 2500 images were annotated across the data collected from three years, with the largest number of annotations being done on the images collected in 2017 as seen in Table 1. It is also shown in the table that a total of four datasets were created, one for each of the harvest years (2015, 2016, & 2017) and a final set that contains all of the data from the three years combined (151617). For each of the datasets, train and test is split randomly at roughly 60% and 40% respectively. The division of years was done to evaluate how a CNN model would react to being trained on images from one harvest with its given conditions and how the resulting model would perform on images from another harvest year. The visual appearance of the crop can change due to the variations in farming such as geographical location, weather conditions, or plant maturity. The combination of data in 151617 is to evaluate the large data requirement of deep learning models and to see if models tuned to specific conditions or a model with larger variation is preferable.

### 2.3. Deep Learning Models

This section covers the two deep learning approaches used for kernel fragment recognition in both object detection and instance segmentation form. First, we will give a short overview of deep learning and CNNs with respect to the core concepts. Deep learning is a form of machine learning that aims to solve a task using a “deep” model through the transformation of data using various functions that can represent the data in a hierachical manner [24]. Deep learning can be especially successful as it allows for automatic feature extraction, rather than an engineer designing hand-crafted features. If the dataset is representative of the deployment scenario it can allow the model to learn a strong set of functions that can be difficult for an engineer to find. However, due to this deep hierarchical manner, the features determined by the model can be difficult to debug and are often treated as a black box. In deep learning the aim is to have model learn a feedforward mapping between input and output, for example, given an input of an RGB image of maize silage output, the x-y coordinates of kernels together with a confidence score of the prediction. In order to learn this mapping the aim is to update the parameters of the model through training to give the desired output. The model is trained over a number of iterations where given the model and its current set of parameters, it perform the feedforward mapping for an image and measures the error of the model in comparison to the correct answer defined in the annotation. This error can then be used to push the model parameters in the correct direction by updating them through the method of backpropagation. Here, the error traverses back through the network and computes the gradient for each function’s parameters that should decrease the error. Using this gradient, an optimisation algorithm, such as Stochastic Gradient Descent (SGD), updates the parameters of the function. This process is continuously performed until the model has updated the parameters in such a way to best perform the mapping of input and output with the lowest possible error in the training set whilst still performing well on a validation set. Depending on the task there are a number of different types of architectures within deep learning: this includes recurrent neural networks often used for natural language processing, reinforcement learning used in robotics, and CNNs used in this case for RGB images. With CNNs the deep hierarchy of functions mainly revolve around the convolution mathematical operation which is well suited for the grid-like topology of images. The convolution operation is relatively simple and has been used in hand-crafted feature engineering such as edge detection or image blurring. Convolution is computed by a filter of a given size (i.e., 3 × 3 or 5 × 5) sliding over the image data and computing an elementwise multiplication and producing a single output value in a feature map. Convolving over the entire image produces a fully realised feature map. The deep aspect of CNNs is therefore a large number of convolution layers computing feature maps upon previously computed maps in succession. The learning process described earlier for CNNs aims to learn the weights in the hierarchy of convolution filters that give the optimal mapping between input and output.

The methods chosen in this work are the Region-based Fully Convolutional Network (R-FCN) [25] for bounding-box detection and the Multi-task Network Cascade (MNC) [26] for instance segmentation. These were chosen due to their state-of-the-art nature at the time of conducting this work, where both performed well on a number of object recognition benchmarks including PASCAL VOC [27] and MS COCO [28].

**Figure 4:**
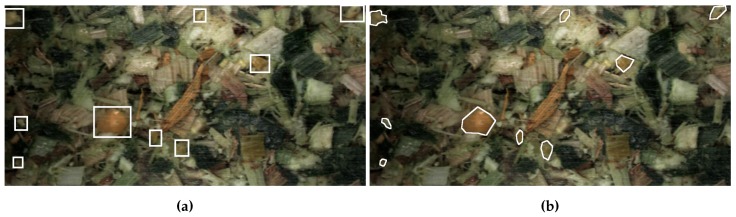
Examples of the difference in localisation granularity between bounding-boxes and segmentation. (**a**) Bounding-box localisation, (**b**) Segmentation localisation. The segmentation localisation fits much closer to the kernel instances and thereby can give a more precise measurement on kernel size.

The CNN approaches solve the task of object recognition but at different degrees of localisation granularity. Bounding-box detectors place an axis-aligned bounding-box around the detected object whereas segmentation indicates the object at a pixel level. Due to the lower localisation granularity of bounding-box detectors, they may over-sample the object and give a larger indication of size than is actually true. This difference on an image from this work can be seen in Figure 4.

This remainder of this section includes an overview of how the methods perform their respective forms of object recognition by covering the model architecture and defining the model and learning parameters used in this work.

#### 2.3.1. Region-Based Fully Convolutional Networks (R-FCN)

**Figure 5:**
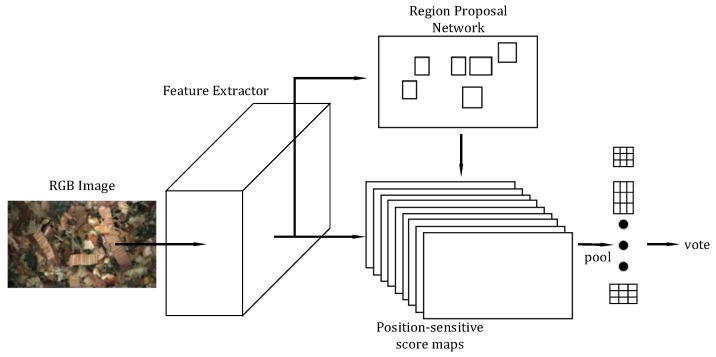
The R-FCN architecture illustrating an image being passed through a number of convolutional layers. RoIs are computed from a RPN on the final convolutional layer, these RoIs are classified through the coloured position-sensitive score maps.

R-FCN is a bounding-box CNN-based object detection method and is based on the popular two-stage detection strategy of object proposals followed by classification of found proposals. Additionally, the authors were one of the first to adapt Fully Convolutional Networks (FCNs) into the two-stage pipeline, rather than using feature pooling layers, such as Region of Interest (RoI) pooling as in the Faster R-CNN detector [29]. Thus, potentially important spatial information is not discarded as can be the case when pooling features. The R-FCN architecture can be seen in Figure 5. In the first stage, an input RGB image is passed through a number of convolutional layers to create a deep representation through a number of feature maps. As is common practice in object recognition through CNNs, the convolutional layers can take many forms that can vary in complexity. Popular choices for the layers include AlexNet [18], VGG [30] and ResNets [31], where in the original R-FCN work the ResNet-101 network was primarily explored. Class-agnostic RoI object locations are found by a Region Proposal Network (RPN) [29]. The RPN finds RoI proposals by sliding a small network over the last feature map computed by the previous convolutional layers. At each sliding window location a number of anchor boxes with varying scales and aspect ratios predict the confidence of a location containing an object. In the second stage, candidate RoI proposal features via an FCN for classification are extracted from a number of position-sensitive score maps. A total of $k^{2}\left(C+1\right)$ maps are computed where *C* is the number of object classes and $k^{2}$ is the spatial grid representing relative positions. In the case shown in Figure 5, *k* = 3, therefore, nine score maps are computed for each object class.

The R-FCNs trained for kernel detection in this work largely follow the same procedure as that conducted in the original work. The network weights were initialised from a pretrained ResNet-101 for ImageNet [32] classification supplied by the authors. The networks were trained for a total of 110,000 iterations using SGD with an initial learning rate of 0.001 and after 80,000 iterations the learning rate was dropped by 0.1. Additionally, momentum of 0.9 and weight decay of 0.0005 was used during optimisation. With respect to the position-sensitive score maps *k* = 3. For each image the mean RGB ImageNet values are subtracted to normalise the training set which aids in the learning process. Subtracting the mean RGB from our training datasets was also evaluated during early development, however, it showed that results were better when using the ImageNet means. Horizontal flipping was the only data augmentation strategy used during training and images were scaled such that the height was 600 pixels and the width was then scaled accordingly to keep the original aspect ratio.

#### 2.3.2. Instance-Aware Semantic Segmentation via Multi-Task Network Cascades (MNC)

**Figure 6:**
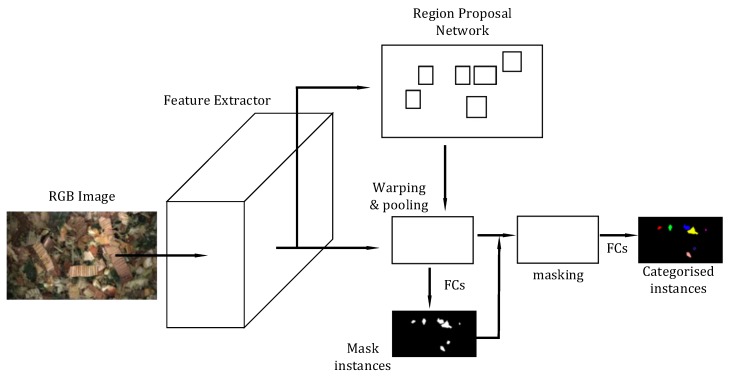
The Multi-task Network Cascade (MNC) architecture, as in Region-based Fully Convolutional Network (R-FCN), an image is passed through a number of convolutional layers and RoIs are found with an RPN. Features are extracted from the RoIs via RoI warping and pooling. Class agnostic masks are founding from the features that are being passed through FC layers. The masks are classified from the RoI features through another set of FC layers.

MNC also follows the mantra of multi-stage object recognition. The task in MNC is instance segmentation where the key difference between R-FCN is a module for determining mask instances, in addition to the region proposals and classification modules. The general architecture of MNC can be seen in Figure 6. As in R-FCN, a feature map is extracted from the last of a number of convolutional layers computed based on an input RGB image. The authors performed their primary experiments using the VGG-16 networks, however, as in R-FCN any popular or user-designed CNN architecture can be used for feature extraction. An RPN determines class-agnostic region proposals followed by RoI warping and pooling. These are used as input to the mask generation modules in combination with learnt fully-connected (FC) layers. Finally, the masks in combination with another set of FC layers perform classification of the mask instances.

As the name implies and as shown in Figure 6, MNC is a cascaded approach for instance segmentation of first determining box instances then mask instances and lastly categorising the instances. However, it is common practice to refine the predictions by extending the cascade to five stages by repeating both the mask generation and classification module. This approach was adapted in this work from the open source code provided by the authors. The work included a pre-trained VGG-16 network trained on ImageNet which was used for transfer learning. However, due to the large complexity of using VGG-16 as a feature extractor, an ImageNet pre-trained AlexNet feature extractor was adapted instead. Following the author’s procedures, MNC models were trained for a total of 25,000 iterations using SGD with an initial learning rate of 0.001. After 20,000 iterations, the learning rate was decreased by 0.1. Additionally, momentum of 0.9 and a weight decay of 0.0005 was used. As in the R-FCN models, the ImageNet RGB mean values were subtracted from the images. Again, horizontal flipping was the only data augmentation implemented and images were scaled such that the height was 600 pixels with width scaled accordingly.

### 2.4. Hardware

Models were trained on an Ubuntu 16.04 machine with an NVIDIA Titan XP Graphics Processing Unit (GPU) using the Caffe framework [33]. Caffe is a deep learning framework developed by Berkely AI Research that allows for fast training of testing of multiple types of models including CNNs and recurrent neural networks. An overview of the memory requirements for training the R-FCN and MNC models and inference speed can be seen in Table 2. While the two models have a relatively low requirement on GPU memory, the difference in the feature extractor can be seen for both train and test memory. The considerably larger and more complex ResNet-101 model present in R-FCN increases the memory usage and adds to the inference timings in comparison to MNC with the AlexNet backbone.

### 2.5. Computer Vision Metrics

Both of the algorithms can be evaluated on an object-level. These metrics do not directly measure how well a prediction intersects with the ground truth instance, rather, it is a measurement of whether or not an instance is correctly classified given a minimum Intersection-over-Union (IoU) threshold between the two. If a prediction overlaps by more than the IoU threshold it can be determined as a true positive detection, otherwise, it is a false positive. In this work an IoU of 0.5 is used when presenting results for the object-level metrics. It should also be noted that only a single prediction can be considered as a true positive with a given ground truth—typically this is the prediction with the highest IoU. If multiple predictions overlap above the threshold, the remaining are considered as false positives.

Firstly, the precision on a dataset can be calculated as:

(1) $$Precision=\frac{TP_{objects}}{TP_{objects}+FP_{objects}},$$

where $TP_{objects}$ and $FP_{objects}$ are the total number of true positives and false positives object instances.

The recall of a dataset is calculated by:

(2) $$Recall=\frac{TP_{objects}}{P_{objects}},$$

where $P_{objects}$ is the total number of positive ground truth examples.

Additionally, Average Precision (AP) is calculated as the mean precision of a dataset and is calculated across 11 equally spaced levels of recall [0, 0.1, ..., 1]. AP is determined by:

(3) $$AP=\frac{1}{11}\sum_{r\in \{0,0.1,...,1\}}\rho interp\left(r\right),$$

where the precision at each level of recall *r* is interpolated by the maximum precision measured for which the corresponding recall exceeds *r*:

(4) $$\rho interp\left(r\right)=\max_{\tilde{r}:\tilde{r}\geq r}\rho \left(\tilde{r}\right),$$

where $\rho (\tilde{r})$ is the measure precision at recall $\tilde{r}$.

The F1-score is calculated by:

(5) $$F1-Score=\frac{2TP_{objects}}{2TP_{objects}+FP_{objects}+FN_{objects}},$$

where $FN_{objects}$ are the total number of non-identified ground truth instances.

## 3. Results

The results for the various trained models according to the metrics defined in Section 2.5 will be covered. Finally, an analysis of Kernel Processing Score (KPS) will be conducted to address the potential of using the system for silage quality evaluation in an industry setting.

### 3.1. Computer Vision Results

**Figure 7:**
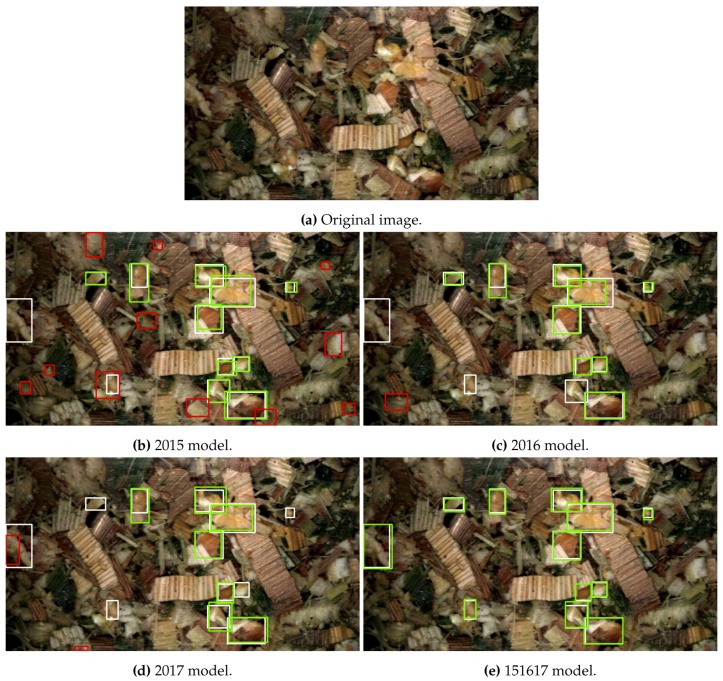
Model predictions on a test image from 2016. Bounding-boxes colours indicate ground truth (**white**), true positive (**green**) and false positive (**red**). True positives and false positives evaluated at an IoU threshold of 0.5.

**Figure 8:**
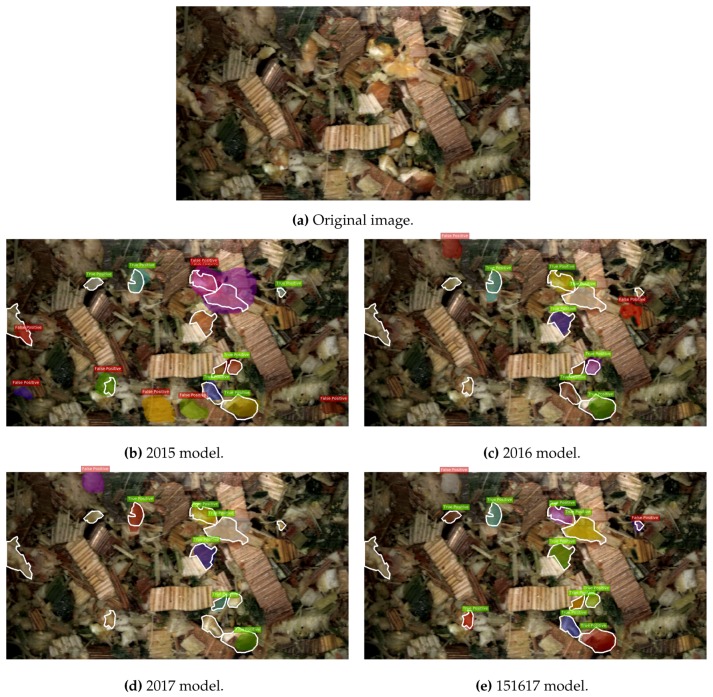
Model predictions on a test image from 2016. Ground truth annotations are shown as a white outline around the kernel fragment. The colour in the text box indicate true positive (**green**) and false positive (**red**). True positives and false positives evaluated at an IoU threshold of 0.5. The individual colour for each prediction indicate separate instances of predictions.

Firstly, detections from the four trained R-FCN models can be seen on an example image from the 2016 test set in Figure 7 and the corresponding four MNC models in Figure 8. In Figure 7b–e the ground truth bounding-box annotations are shown in white around the kernel fragments, true positive detections are shown in green, and false positives are shown as red. Whereas in Figure 8b–e the annotations are shown as a white outline around the kernel fragment, individual kernel fragment instance predictions are shown with different colours, while the determination of true positive or false positive is indicated by the green or red text above the prediction. In both figures, detections were considered as either a true positive or false positive at an IoU threshold of 0.5. The original image can be seen in Figure 7a and Figure 8a.

An overview of the metrics covered in the previous section are shown below for the models trained and tested on the respective datasets defined in Table 1. As stated in Section 2.3.1 and Section 2.3.2, the four respective R-FCN and MNC models were trained using a consistent architecture and learning parameters. The only difference is the training dataset itself, where the content aimed to give an insight into the varying field conditions in agriculture from harvesting season to season. Additionally, there is a considerable difference in the amount of data annotated in the sets, where the 2017 sets have around 10× more images in both training and testing. Of course, this also has the effect of images captured in 2017 being the significant majority in the combined 151617 dataset. An overview of the results for the computer vision metrics can be seen in Table 3. For each test set the best performing model for a given metric is shown in bold. The general trend seen in the table is that the 151617 model is the most robust across the four test sets, in many cases being the best performing for a metric or the second best. The differences between then R-FCN and MNC models are slight with only a few percentage points difference for all test sets apart from the 2015 test set. For this test set the model trained on the larger 151617 dataset performs considerably better than the other models across all metrics, including the 2015 model which is trained on only images from the same year as the test set. The R-FCN 151617 model achieves a 65.9% AP, 31.9% points higher than that of the 2015 counterpart. Whereas the AP for the MNC model is significantly lower at 40.4%, a significant increase is still present compared to the 2015 MNC model. Additionally, for the 151617 R-FCN model precision and recall scores at 70.0% and 76.0%, roughly 20.0% points higher than the 2015 model in both regards. The considerable improvement of the 151617 model in comparison to 2015 is present despite images from 2015 only making up around 10% of the training material in 151617. However, this 10% in addition to the roughly 10% from 2016 seems to have a significant impact as the model trained on data only from 2017 performs worse than both 2015 and 151617 models at 28.5% AP.

As mentioned, the difference in results between R-FCN and MNC models are not as significant for the remaining test sets, however, the trend of the combined 151617 training dataset giving robust results continue. The 151617 models is the best performing for both models by considerable margins. AP for the 151617 model scores at 66.9% and 71.8% for R-FCN and MNC respectively, 25.1% and 19.7% points higher than the 2016 models. Similar increases in the remaining metrics exist as of that for the 2015 test set. Once again images similar to the test set is in the minority in the 151617 training set with around 10% being harvested in 2016. As in the results for the 2015 test set this 10% addition has a considerable effect as the relatively large 2017 model is the third best performing model on most metrics.

The 2017 and 151617 results do not show an as significant difference in the results as for 2015 and 2016. The best performing model varies across the numerous metrics, however, the 2017 and 151617 models measure consistently well in comparison to the other two who lack in some regards. For example, the 2015 R-FCN model has a relatively high recall of 70.5% but poorer precision of 19.0%. Whereas the 2016 R-FCN model has the highest precision on both 2017 and 151617 test sets at 43.4% and 50.1%, however, the AP is considerably lower at around 10% points. The results are similarly not as varying for the MNC models, with the 2017 and 151617 models in general performing strongest. In general, there is negligible difference between the 2017 and 151617 models for both R-FCN and MNC on the corresponding two test sets. This is likely because the training set between the two models has much more overlap than the earlier results.

### 3.2. Kernel Processing

**Figure 9:**
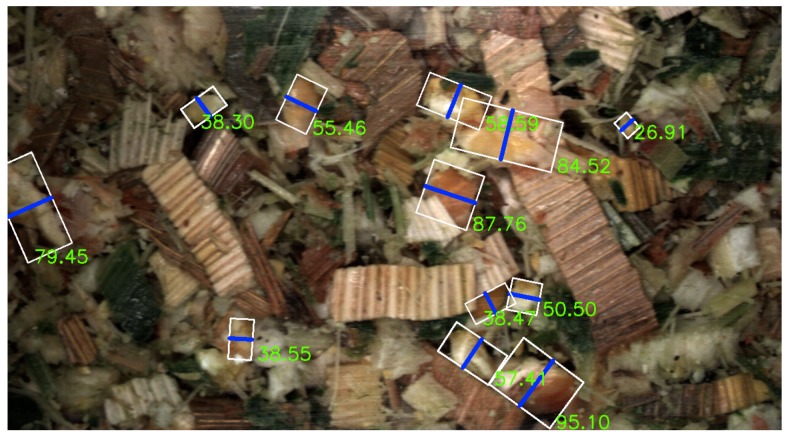
Visualisation of determining kernel processing based on the shortest axis length of a rotated bounding-box for a number of annotated kernel fragments. The shortest axis is shown via a blue line with the length in pixels for each shown next to the fragment.

To evaluate the viability of the two CNN methods for kernel fragment recognition, we adopt the commonly used KPS score from the CSPS [3]. For each detected instance for either method the length of smallest axis from a rotated fitted bounding-box is found. This length gives an indication of the detected kernel instance that would pass through the 4.75 mm sieve screen used in CSPS. The smallest axis length is used as a quality indicator due to the three-dimensional shaking present in the Ro-Tap separators used in CSPS, therefore, particles are separated based upon the shortest diameter. The KPS was also used to evaluate the image processing algorithm developed in [6], however, the diameter of the largest inscribed circle was used in this case. Additionally, in [6] the actual KPS was calculated by performing the Ro-Tap laboratory separation, unfortunately, this was not done while harvesting in this work. Instead we calculated the KPS for a sequence of images from a given PG by determining the shortest axis length from the ground truth annotations. As the images of the silage were taken with known distance to the camera, the pixel resolution in mm can be converted as 1 mm to 20 pixels, meaning that kernels of lengths below 95 pixels (4.75 mm) are considered to be optimally processed. Figure 9 shows the calculation of the minor axis from a rotated bounding-box for an annotated image. In this example a single kernel is above the 4.75 mm threshold and deemed not optimally processed with a minor axis of 95.10 pixels.

Due to the large number of annotations present for images from 2017, a number of different sequences were created with different conditions. This is shown in the left-most two columns in Table 4, with 17 sequences with varying PGs. The table also shows the KPS calculated as the percentage of kernel fragment detections with a shorter axis below 4.75 mm for the eight respective models trained on different subsets of data. It should be noted that because of the nature of the predictions between the R-FCN and MNC models, it was only possible to determine a rotated bounding box for the MNC predictions due to the higher localisation granularity of pixel-level segmentation. Instead, for the R-FCN detections, the shortest distance of the axis-aligned bounding box was taken. Finally, the KPS ground truth from the annotations is shown in the right-most column. The average absolute error summarises the accuracy of each model of all PGs in the final row. While there are individual differences in the KPS calculation in comparison to the annotations from different sequences, in general the average absolute error is lowest for the 151617 R-FCN model—4.5% points less than the MNC counterpart despite having the disadvantage of axis-aligned bounding-boxes.

### 3.3. Correlation Analysis

Given the results in Table 4 across the varying PGs, the effectiveness of the KPS calculation can be evaluated across a number of different potential sizes of kernel fragments. This section covers a correlation analysis for both the R-FCN and MNC method.

#### 3.3.1. R-FCN

**Figure 10:**
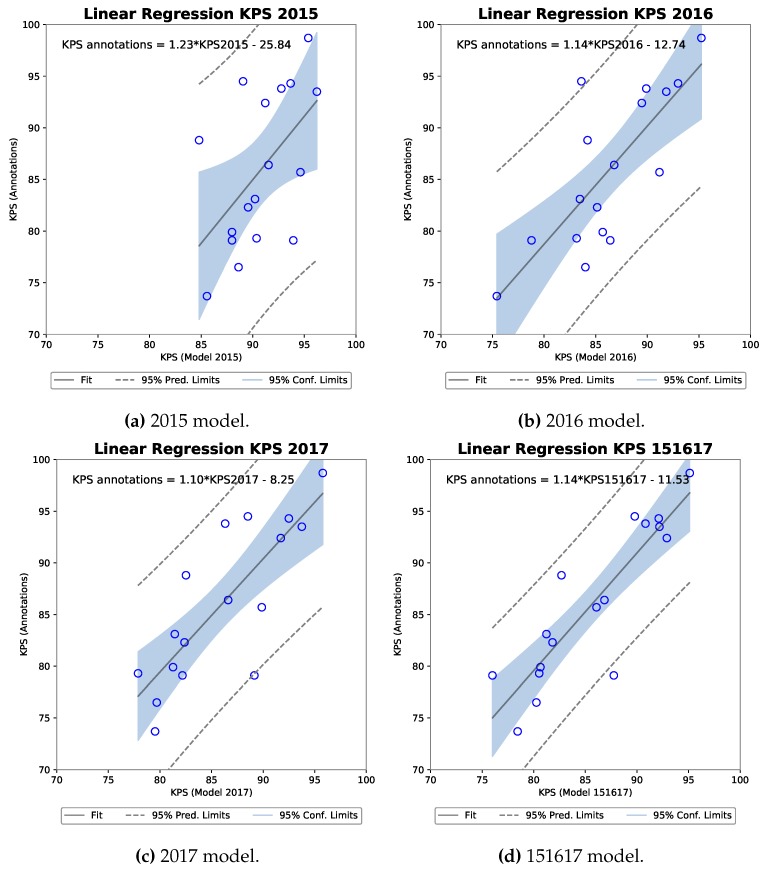
Four scatter plots of the R-FCN model KPS against annotation KPS with linear regression analysis computer for each.

Four scatter plots including the equation describing the linear regression fit can be seen for each R-FCN model against the KPS annotations in Figure 10. Each indicate a positive slope of an increasing KPS for a model as the ground truth KPS increases.

To determine the significance of a potential correlation, a Pearson’s correlation coefficient was calculated as shown in Table 5. Results from a Shapiro-Wilk normality test are also shown, as Pearson’s assumes that both samples arise from a normal distribution. A high W, as present for all five samples in Table 5, means that the null hypothesis that the population is normally distributed cannot be rejected. Following [34] we can interpret the results for Pearson’s correlation coefficient that all models have a strong positive correlation to the annotation KPS. The strongest being the 151617 model of r(15) = 0.88 with a *p*-value of 0.000003, explaining 77.7% of the variance in the ground truth KPS.

#### 3.3.2. MNC

**Figure 11:**
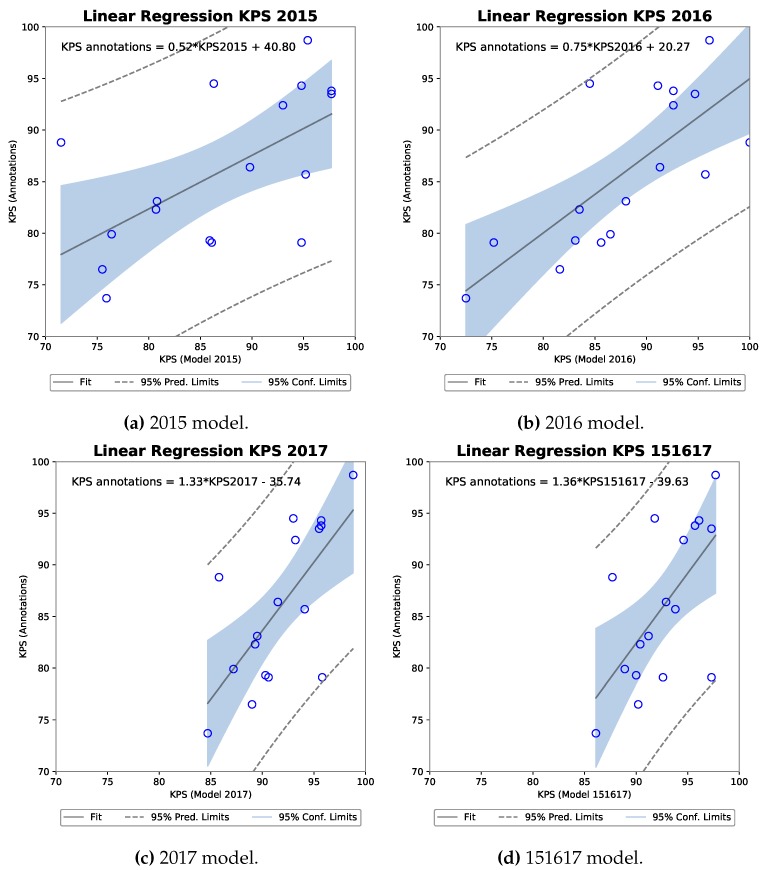
Four scatter plots of the MNC model KPS against annotation KPS with linear regression analysis computer for each.

The corresponding four scatter plots for the MNC models can be seen in Figure 11. Again, a positive relationship is indicated between the KPS from each model and the KPS for annotations across processor gaps.

Table 6 firstly show Shapiro-Wilk tests for each sample with high W and corresponding p-values. The resulting Pearson’s correlation coefficient also indicates a strong positive correlation. The strong appears from the 2016 model with r(15) = 0.74 with a *p*-value of 0.0007, explaining 54.4% of the variance in the KPS annotations.

## 4. Discussion

The potential to train CNN models for kernel fragment recognition in RGB images of silage is promising. This appears to be the case even without conducting the time-consuming step of separating kernels and stover before evaluation, as in all current popular kernel fragmentation evaluation methods [3,4,5,6].

The four models trained in both R-FCN bounding-box and MNC instance segmentation performed well and two major tendencies appeared. Firstly and possibly unsurprisingly, a larger training dataset, such as that of 151617, led to models that performed well across all metrics on all test sets. Deep learning methods are known to have a high requirement on the amount of data and the roughly 10× larger 151617 training set in comparison to the 2015 and 2016 sets seemed to show this effect. However, a total of 1393 images with 6907 annotated kernel instances is not on the same level as considerably larger object recognition benchmarks such as PASCAL VOC [27] or MS COCO [28] consisting of over 10,000 and 165,000 images for training respectively. The trained R-FCN and MNC models of course take advantage of transfer learning from a pre-trained models on ImageNet datasets. With this aid, roughly 1400 annotated training images in 151617 set gave consistent results across test images from three different harvest years. Additionally, the second finding was of the at times significant improvement when adding only a small amount of data to a larger dataset. This was seen for the models trained on the 151617 dataset for test sets 2015 and 2016, where despite the additional data being in the minority during training in contrast to images from 2017, they had a large increase in performance compared to models that did not combine all of the data.

With respect to the viability of using a CNN-based model for KPS measurement, both methods can be deemed to have potential. A strong positive correlation was found between annotation KPS and model KPS, with the strongest existing for the 151617 R-FCN model. A criticism of the correlation analysis is naturally that this was against annotation KPS and not a truer laboratory measurement than in [6]. However, as the training and testing splits were kept separate, the correlation results still give a good indication for the approaches.

In comparison to [6] who show KPS measurement given manually separated kernels in a controlled camera setting, the error measurement across sequences is similar to our work. KPS based on image analysis from wet samples from the field from [6] show an average absolute error of 5.6% in comparison to our range of 2.7% to 7.2% dependent on the model and test set. Of course, care should be taken comparing the two works given the differences in ground truth measurement, location of harvesting, the machine, and so forth. A key improvement in this work is the time required to obtain a KPS measurement. In [6] the time was improved to hours instead of days as in [3], however, due to removing the requirement of kernel/stover separation, this work allows KPS calculation to be done in minutes.

Future work is to evaluate against a laboratory measured KPS as mentioned earlier. Furthermore, research into applying newer object recognition methods from the fast-moving field may also be viable, potentially improving challenges such as recognition of small objects. Finally, such CNN-based methods could be used to measure other silage-quality aspects, such as the cutting length of the forage harvester.

## 5. Conclusions

This work has shown that kernel fragmentation in maize silage can be estimated from images using trained CNNs in both bounding-box and instance segmentation form. Through transfer learning and training models on images captured across three different harvest seasons, both forms were able to estimate the fragmentation robustly. This was evaluated via computer vision metrics and an analysis of the correlation between model predictions and a kernel processing score. Where the latter showed a strong correlation for both CNN forms to an industry standard kernel processing score.

Furthermore, this work showed promise in kernel fragmentation estimation in non-separated kernel/stover images, leading to a potentially significant decrease in measurement time.

## Figures and Tables

**Table 1:** Overview of datasets created based on the year in which the images were captured. The total number of images and kernel instances per dataset is shown.

	2015	2016	2017	151617
Train Images	111	115	1167	1393
Train Kernel Instances	1388	675	4844	6907
Test Images	76	85	884	1045
Test Kernel Instances	836	433	3425	4694

**Table 2:** Overview of hardware statistics for both methods. Timings were done on images of size 600 × 1000 pixels on an Ubuntu 16.04 machine with an NVIDIA Titan XP GPU.

	Train Memory (MB)	Test Memory (MB)	Inference Time per Image (s)
**R-FCN (ResNet-101)**	6877	3251	0.101
**MNC (AlexNet)**	3439	2369	0.087

**Table 3:** Computer vision metric results for both the R-FCN and MNC models across the four test sets.

			R-FCN				MNC	
**Train Dataset**	**AP**	**Prec**	**Recall**	**F1-Score**	**AP**	**Prec**	**Recall**	**F1-Score**
**2015 Test**								
2015	34.0	55.5	53.0	54.2	27.7	44.5	31.8	37.1
2016	19.0	**80.0**	21.0	33.3	16.8	60.5	16.5	25.9
2017	28.5	51.1	40.2	45.0	27.7	50.0	32.1	39.1
151617	**65.9**	70.0	**76.0**	**73.9**	40.4	50.3	46.3	48.2
**2016 Test**								
2015	25.3	23.3	87.1	36.8	40.7	30.1	61.0	40.3
2016	41.8	52.1	73.2	60.9	52.1	54.4	62.8	58.3
2017	34.2	41.7	63.1	50.2	53.0	45.7	67.9	54.6
151617	66.9	**56.9**	**90.8**	**70.0**	**71.8**	47.6	80.8	59.9
**2017 Test**								
2015	15.3	19.0	**70.5**	29.9	18.6	20.2	36.4	25.8
2016	19.2	**43.4**	44.1	43.7	24.3	39.8	32.8	36.0
2017	31.0	36.4	66.9	47.2	**36.3**	32.9	53.3	40.7
151617	33.4	37.6	**67.2**	**48.2**	35.9	31.9	53.7	40.0
**151617 Test**								
2015	19.6	23.4	**73.6**	35.6	26.1	26.2	42.9	32.5
2016	22.3	**50.1**	44.7	47.2	28.4	46.7	34.2	39.5
2017	30.2	39.2	62.5	48.2	35.8	36.0	51.0	42.2
151617	34.0	40.7	66.0	**50.4**	**36.1**	34.2	52.2	41.4

**Table 4:** Kernel Processing Score (KPS) results across sequences of varying PGs for R-FCN and MNC models. The final row shows the average absolute error for each model over all sequences from the 2017 test set.

%(<4.75 mm)	2015		2016		2017		151617		
**PG**	**R-FCN**	**MNC**	**R-FCN**	**MNC**	**R-FCN**	**MNC**	**R-FCN**	**MNC**	**Annotation**
1	96.2	97.7	91.8	94.7	93.8	95.5	92.2	97.3	93.5
1	95.4	95.4	95.2	96.1	95.8	98.8	95.1	97.7	98.7
1	88.0	76.4	85.7	86.5	81.3	87.2	80.7	88.9	79.9
1	93.7	94.8	93.0	91.1	92.5	95.7	92.1	96.1	94.3
2	93.9	94.8	78.8	75.2	89.2	95.8	87.8	97.3	79.1
2	92.8	97.7	89.9	92.6	86.3	95.7	90.8	95.7	93.8
2	84.8	71.5	84.2	100.0	82.5	85.8	82.7	87.7	88.8
2	88.0	86.1	86.4	85.6	82.2	90.6	76.0	92.6	79.1
3	89.6	80.7	85.1	83.5	82.4	89.3	81.8	90.4	82.3
3	94.6	95.2	91.2	95.7	89.9	94.1	86.1	93.8	85.7
3	90.4	85.9	83.2	83.1	77.9	90.3	80.5	90.0	79.3
3	89.1	86.3	83.6	84.5	88.5	93.0	89.8	91.8	94.5
3.5	90.2	80.8	83.5	88.0	81.4	89.5	81.2	91.2	83.1
3.5	88.6	75.5	84.0	81.6	79.7	89.0	80.3	90.2	76.6
3.5	91.2	93.0	89.5	92.6	91.7	93.2	92.9	94.6	92.4
3.5	85.6	75.9	75.4	72.5	79.5	84.7	78.4	86.1	73.7
3.5	91.5	89.8	86.8	91.3	86.6	91.5	86.9	92.9	86.4
Avg. abs. error	6.7	5.3	3.8	4.6	3.3	6.3	2.7	7.2	

**Table 5:** Correlation analysis via Pearson’s correlation coefficient for the KPS of the four R-FCN models against the annotation KPS. Pearson’s assumes a normal distribution in the data which is evaluated through a Shapiro-Wilk normality test.

	Shapiro-Wilk		Pearson’s Correlation		
**KPS**	**W**	**p-value**	**r(15)**	**p-value**	**$r^{2}$ (%)**
Annotations	0.94	0.32	NA	NA	NA
2015	0.973	0.870	0.54	0.0244	29.4
2016	0.97	0.816	0.77	0.0003	59.5
2017	0.94	0.320	0.81	0.00009	65.1
151617	0.94	0.327	0.88	0.000003	77.7

**Table 6:** Correlation analysis via Pearson’s correlation coefficient for KPS of the four MNC models against the annotation KPS. Pearson’s assumes a normal distribution in the data which is evaluated through a Shapiro-Wilk normality test.

	Shapiro-Wilk		Pearson’s Correlation		
**KPS**	**W**	***p*-Value**	**r(15)**	***p*-Value**	**$r^{2}$ (%)**
Annotations	0.94	0.32			
2015	0.91	0.098	0.60	0.0106	36.2
2016	0.97	0.743	0.74	0.0007	54.4
2017	0.97	0.806	0.69	0.002	48.1
151617	0.97	0.666	0.63	0.0065	39.9

## Abbreviations

The following abbreviations are used in this manuscript:

RGB	Red, Green, and Blue
PG	Processor Gap
CSPS	Corn Silage Processing Score
PSPS	Penn State Particle Separator
CNN	Convolutional Neural Network
CL	Cutting Length
R-FCN	Region-based Fully Convolutional Network
MNC	Multi-task Network Cascades
FCN	Fully Convolutional Network
RoI	Region of Interest
RPN	Region Proposal Network
SGD	Stochastic Gradient Descent
FC	Fully-Connected
GPU	Graphics Processing Unit
KPS	Kernel Processing Score
IoU	Intersection-over-Union
AP	Average Precision
